# Phase I study of patients with non-muscle invasive bladder cancer (NMIBC) treated with vesigenurtacel-L (HS-410) after Bacillus Calmette-Guérin (BCG)

**DOI:** 10.1186/2051-1426-3-S2-P447

**Published:** 2015-11-04

**Authors:** Gary Steinberg, Neal Shore, Lawrence Karsh, James Bailen, Michael Woods, Taylor Schreiber, Melissa Price

**Affiliations:** 1 University of Chicago Medical Center, Chicago, IL, USA; 2 Atlantic Urology Clinics, Myrtle Beach, SC, USA; 3 The Urology Center of Colorado, Denver, CO, USA; 4 First Urology, Jeffersonville, IN, USA; 5 The University of North Carolina at Chapel Hill, Chapel Hill, NC, USA; 6 Heat Biologics, Inc., Durham, NC, USA

## Background

Vesigenurtacel-L (HS-410), consists of an allogeneic bladder cancer cell line, selected for high expression of a series of tumor antigens that are known to be shared by a high proportion of bladder tumors. The cell line secretes gp96-Ig, a modified heat shock protein which delivers cell-derived antigens to MHC-I via the cross-presentation pathway, leading to preferential activation of CD8+ cytotoxic T cells. Vesigenurtacel-L was evaluated in a Phase I trial after treatment with standard of care induction BCG for safety and immune response.

## Methods

Ten patients with non-muscle invasive bladder cancer who had undergone TURBT, were judged to be at an increased risk for recurrence, and were either BCG naïve or had completed previous BCG treatment >12 months prior to the most recent TURBT were treated with induction BCG as standard of care and enrolled in the trial. Patients received up to 15 doses of monotherapy vesigenurtacel-L at a dose of 10^6^ cells per dose, weekly for 12 weeks followed by 3 monthly doses. Baseline tumor tissue and any follow up biopsies were evaluated by immunohistochemistry for tumor infiltrating lymphocytes and expression of PD-L1.

## Results

Vesigenurtacel-L was well tolerated with no treatment-related Grade 3/4 adverse events. The most common adverse events were low-grade injection site reactions consistent with delayed type hypersensitivity. Peripheral blood mononuclear cells were evaluated by flow cytometry for detection of circulating leukocyte subsets, regulatory T cells, myeloid derived suppressor cells, activated T cells and expression of immune checkpoint molecules on T cells. Additionally, analyses from pre- and post-treatment tissue specimens in a subset of patients including antigen expression, evaluation of tumor-infiltrating lymphocytes, PD-L1 expression and T cell receptor sequencing will be reported, and their extrapolation to an emerging definition of responder phenotype discussed. For example, tissue analysis indicates an increase in CD8+ T cells in the bladder after treatment, consistent with the mechanism of action derived from preclinical models, and this change appears be correlated to clinical outcome.

## Trial registration

Clinical trial information: NCT02010203.

